# In Vitro and In Vivo Potential of RH Strain of *Toxoplasma gondii* (Type I) in Tissue Cyst Forming

**Published:** 2013

**Authors:** Qasem ASGARI, Hossein KESHAVARZ, Saeedeh SHOJAEE, Mohammad Hossein MOTAZEDIAN, Mehdi MOHEBALI, Ramin MIRI, Davood MEHRABANI, Mostafa REZAEIAN

**Affiliations:** 1 Dept. of Medical Parasitology and Mycology, School of Public Health, Tehran University of Medical Sciences, Tehran, Iran; 2 Center for Research of Endemic Parasites of Iran, Tehran University of Medical Sciences, Tehran, Iran; 3 Dept. of Medical Parasitology and Mycology, Faculty of Medicine, Shiraz University of Medical Sciences, Shiraz, Iran; 4 Medicinal and Natural Products Chemistry Research Center, Shiraz University of Medical Sciences, Shiraz, Iran; 5 Stem Cell and Transgenic Technology Research Center, Shiraz University of Medical Sciences, Shiraz, Iran

**Keywords:** *Toxoplasma gondii*, RH strain, Tissue cyst, in vitro, in vivo

## Abstract

**Background:**

Based on recent studies, there are controversial reports on the capacity of tissue cyst forming of *Toxoplasma gondii* RH strain. In this study, the capacity was evaluated by *in vivo* and *in vitro* experiments.

**Methods:**

RH strain was subcutaneously inoculated to ten Wistar rats. After one month, their blood, brain, tongue and diaphragm were collected and evaluated by MAT, PCR, pathological and bioassay methods. The parasite was cultivated in the cell monolayer. To change to bradyzoite, the media pH was altered to 6.8. Biological aspect of the bradyzoites was evaluated by incubation in acidic pepsin and it's inoculation in ten BALB/c mice.

**Results:**

All rats showed antibodies to *Toxoplasma* at titers ≥1:320 but no DNA and tissue cyst were detected in the tissues. Following intraperitoneal inoculation of rats’ brain homogenate into BALB/c mice, no infection was established in none of the animals. During presence of cell culture, in acid media for a 3-5 days period, cyst-like structures were noticed when they were stained with PAS. The visible bradyzoites in the cysts that were incubated in acid pepsin medium were not able to kill any mice.

**Conclusion:**

This study confirmed that Iranian RH strain has lost the potential of tissue cyst forming in rats and bradyzoites cultivated in cell culture lost their resistance to acidic condition, so this strain can be a candidate for future vaccine researches.

## Introduction

*Toxoplasma gondii* as an obligate intracellular protozoan can infect man and a wide range of warm-blooded animals (1). Infection in human hapens due to ingestion of oocysts from the feces of contaminated cats (2, 3) and by ingestion of raw or under-cooked tissue cyst containing products (4, 5). Tachyzoites of *T. gondii* were noticed in dairy products of cows, sheep, goats, cats and mice (6–8).

Mice were demonstrated as animal model of toxoplasmosis due to sensitivity to the disease (9). Rats were reported as a resistant host model and were shown to be a suitable animal model for human toxoplasmosis (10). In the last two decades, cell culture systems have also been introduced as an alternative for animal models reducing the costs and ethical limitations (11) that were intended for diagnostic assays, vaccine strategies, drug sensitivity tests and other proposes such as in biochemistry, genetics and immunology (12, 13).

Isolation of RH strain of *T. gondii* genotyp I in a 6-year-old boy was first reported in 1939 (14) and has been passaged in mice and cell culture in many laboratories worldwide (15). The genotype I of *T*. *gondii* was shown to be responsible for lethal infections in outbred mice while types II and III were significantly less virulent (16). Due to the prolonged passage of this strain, its pathogenicity was stabilized in mice (17), while this strain lost its potential to produce oocysts in cats (18). There are still controversies on the potential of cyst formation of this strain (19–21). It was shown that the RH strain lost its potential to tissue cyst formation in rats due to long passage time of tachyzoites (22). However, a report revealed that in mice, atovaquone together with pyrrolidinedithiocarbamate could change RH tachyzoites of *T. gondii* into tissue cysts (23). In Iran, some researchers presented evidences for tissue cyst formation of this strain in rat (24, 25).

This study determines the in vitro and in vivo potential of RH strain of *T. gondii* (Genotype Ι) in tissue cyst forming in rats.

## Materials and Methods

### Animals

Inbred BALB/c mice (6-8 weeks old weighted 22-25 grams) were provided from Pasteur Institute, Tehran, Iran. Two months old male Wistar albino rats (weighing 150-180 g) were obtained from the Laboratory Animal Center of Shiraz University of Medical Sciences, Shiraz, Iran. Animals were housed in cages and maintained under controlled conditions (21±2°C, 65-70% humidity and standard food and water ad libitum) during the experiments. The experiments were undertaken based on guidelines of laboratory animals in research and teaching book (26).

### Parasite

The virulent RH strain of *T. gondii* was obtained from Tehran University of Medical Sciences, Tehran, Iran. Tachyzoites of this strain were collected by serial intraperitoneal passages in BALB/c mice. Parasites (1×10^5^) were inoculated in the mice, and after 72 hours, tachyzoites were provided by repeated flushing of the peritoneal cavity by Phosphate Buffered Saline (PBS). Tachyzoites were then harvested and centrifuged at 200 g for 5 min at room temperature to remove peritoneal cells and cellular debris. The supernatants were collected and centrifuged at 800 g for 10 min (21). The pellets, enriched with parasite tachyzoites, were recovered with PBS and used in the experiments.

### Inoculation of parasite into rats

The tachyzoites (1×10^5^) of the virulent RH strain were subcutaneously inoculated to 10 albino Wistar rats. After one month, blood, brain, tongue and diaphragm tissues were evaluated by Modified agglutination test (MAT), Polymerease chain reaction (PCR), pathological and bioassay methods.

### Modified agglutination test (MAT)

Blood samples of the animals were collected and the sera were separated. The MAT was performed using formalin fixed whole tachyzoites and mercaptoethanol as previously described Dubey and Desmonts in 1987. A positive reaction at a 1:20 dilution of sera was indicative of previous exposure to the parasite (27).

### Histological examination

To confirm the presence of tissue cyst of the parasite in brain, tongue and diaphragm of rats, the animals were euthanized by ketamine and xylazine. Sampling was undertaken via autopsy and fixed in 10% buffered formalin for histological evaluation stained by hematoxylin and eosin at 100x and 400x magnifications.

### Bioassay in mice

The brain tissues from rats were first homogenized and then intraperitoneally inoculated into 5 mice. If the mice died, their liver impression smears were stained by Giemsa dye and investigated under light microscopy for parasite detection. One month after the inoculation, crush and impression smears were prepared again from liver, spleen and brain tissues of live mice and were parasitologically examined for presence of tachyzoites or tissue cysts.

### DNA extraction

A total of 30 samples were taken from brain and diaphragmatic tissues of the rats. For extraction of DNA, 200 mg of these organs were homogenized and then diluted with double-distilled water (1:10). Proteinase K (10 µL) and lysis buffer (50 ml of Tris–HCl, pH = 7.6; 1 mM of EDTA, pH = 8.0; 1% Tween 20) were added to 500 µl of each sample and the samples were incubated for 24 h at 37°C. The lysate was extracted with phenol/chloroform/isoamylalc-ohol and then with chloroform/isoamylalcohol solutions. The DNA was precipitated with absolute ethanol and then was resuspended in 100 µL of double-distilled water and stored at 4°C until use.

### Nested PCR

Nested primer sets (Bioneer, Korea) were used for amplifying fragments of the B1 gene as previously described (28). The external primers were 5′-GGA ACT GCA TCC GTT CAT GAG-3′ and 5′-TCT TTA AAG CGT TCG TGG TC-3′ producing an amplified product of 193 bp.

All the PCR reactions were performed in a programmable thermocycler (Eppendorph, Mastercycler gradient). The first 25 µL of PCR reaction mixture contained outer primers at a final concentration of 50 pmol each, 2.5 mmoldNTPs, 1 µg of template, and 1.5 U recombinant taq DNA polymerase (GENET BIO, Korea, A-type prime TaqTM DNA polymerase), in 1× PCR reaction buffer (50 mmol/L KCl and 10 mmol/L Tris–HCl, 1.5 mmol/L MgCl2, and 0.1% triton X-100; Sinagen Co., Iran). The first step of amplification was 5 min of denaturation at 94°C. This step was followed by 40 cycles, with one cycle consisting of 10 seconds at 94°C, 10 seconds at the annealing temperature (57°C) for each pair of primers, and 30 seconds at 72°C. The final cycle was followed by an extension step of 10 min at 72°C. Nested reactions contained 1 µl of the first-round product, 10 mMTris–HCl at pH 8.3 and 25°C, 50 pmol each, 2.5 mmoldNTPs, 1 µg of template, and 1.5 U recombinant taq DNA polymerase. Internal primers were 5′-TGCATAGGTTGCAGTCACTG-3′ and 5′-GGCGACCAATCTGCGAATACACC-3′ producing an amplified product of 96 bp. Nested PCRs cycled 40 times using a denaturation step of 93°C for 10 seconds, followed by annealing at 62.5°C for 10 seconds and extension at 72°C for 15 seconds. Negative control samples from first-round amplification and an additional second-round negative control of sterile water were included in the nested reactions. The extracted DNA from RH strain of *T. gondii* was utilized as positive control. The amplification products were detected by gel electrophoresis using 2% agarose gel in 1× Tris–borate–EDTA buffer. DNA bands were visualized in the presence of ultraviolet light, following the staining with 0.5% ethidium bromide.

### Cell culture

HeLa Cells obtained from the Immunology Department, Shiraz University of Medical Sciences, were grown in 5 ml of culture medium using 25 cm^2^ flasks (Corning Costar UK, UK). A monolayer of the cells were provided in RPMI_1640_ with 10% heat-inactivated fetal calf serum (FCS; Gibco Company, USA) and 100 IU/ml penicillin–100 µg/ml streptomycin (Roche Company) and incubated in 5% CO_2_ at 37 °C and >80% humidity. Cells were routinely subcultured every 3 days by trypsination and washing with phosphate-buffered saline (pH = 7.2).

### Cell culture of Toxoplasma gondii tachyzoites

After 70% confluency of the cell line in a monolayer condition, the tachyzoites were added to the cell culture in a ratio 3 to 1. The flasks were then incubated at 37°C, >80% humidityand 5% CO_2_ for 24 h.

### Toxoplasma gondii bradyzoite and tissue cyst formation in cell culture

After 70% confluence of the cell monolayer of the line, pH of the media was reduced to 6.8. Then *T. gondii* RH strain tachyzoites, in a ratio 3 to 1 were added to the cell culture. After 8 hours, when active tachyzoites had entered into the cells, the media were replaced. Afterwards, the media were replaced and their pH media were adjusted every 48 hours.

### Periodic Acid Schiff (PAS)

After removal of the infected cell culture media, the bottom of the flask was cut and dried. Then it was fixed in absolute methanol, allowed to dry, oxidized in 0.5% periodic acid solution for 5 minutes, rinsed in distilled water, placed in Schiff reagent for 15 minutes (changed into light pink color), washed in warm tap water for 5 minutes (changed into dark pink color), counterstained in Mayer's hematoxylin for 1 minute****, washed in tap water for 5 minutes and finally dehydrated and covered with a slip using a synthetic mounting medium.

### Infectivity of tachyzoites and bradyzoites grown in cell culture

The 1×10^5^tachyzoites obtained from the cell culture were intraperitoneally inoculated into 5 mice. As control group, the same number of parasites obtained from intraperitoneal passaging in BALB/c mice was intraperitoneally inoculated into another five mice. In the other experience, on the 5^th^ day, the supernatant of the flask was removed and the precipitate was washed with phosphate-buffered saline (pH = 7.3) and centrifuged at 1200 rpm for 10 min. Three milliliter of acid pepsin solution (pepsin, 2.6 g; NaC1, 5.0 g; HCI, 7.0 ml and distilled water to make 500 ml, pH ∼ 1.10-1.20) as described before (29) was added to the sediment, incubated at 37°C for 60 min and then the homogenate was neutralized with 1.2% sodium bicarbonate. One milliliter of the neutralized homogenate was then intraperitoneally inoculated into each of the five mice.

## Results

After one month of inoculation by RH strain of *T. gondii*, all rats showed antibodies titer ≥1:320. Histologically, no tissue cyst was observed in their brain, tongue and diaphragmatic tissues. Also, no DNA of the parasite was detected in these organs and inoculation of the brain tissue homogenate could not kill any of the BALB/c mice.

**Fig. 1:**
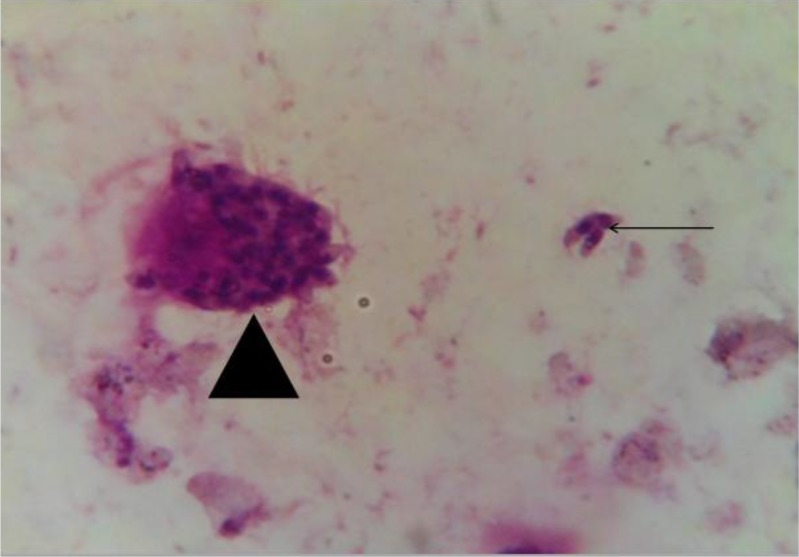
The smear of infected Hela cells by RH strain of *Toxoplasma gondii* due to a change in pH of the medium (The extracellular and the intracellular bradyzoites were arrowheaded; PAS staining, 400X)

In necropsy of the mice, neither tachyzoite nor tissue cyst were visible in crush smears of their brain and liver tissues. During presence of cell culture, in acid media for a 3-5 days period, cyst-like structures were noticed when they were stained with PAS. Figure 1 shows the organisms in red color, demonstrating several amylpectin granules in the organism. After incubation of the bradyzoites in acid pepsin solution and inoculation into BALB/c mice, all mice were still alive and the cyst and bradyzoites in the cell culture were all sensitive to acid-pepsin based on bioassay in mice.

## Discussion

In this study, no tissue cyst and even DNA parasite were detected in brain, tongue and diaphragmatic tissues of rats. In addition, infection of mice was not successful after inoculation of a homogenate of brain tissue of the infected rats into the mice. Our findings were indicative of the fact that the RH strain of *T. gondii* was not capable of any tissue cyst formation in rats but could lead to high titers of antibodies against the parasite. It seems that the immune system responses results into removal of the parasite from the whole body of the host.

The authors in another study (Unpublished data) showed that when RH strain of the parasite tachyzoites were inoculated into rats treated with cyclophosphamide, all animals died within 5-7 days and tachyzoites could be recovered from the autopsy specimens. The virulence of the parasite in a host is dependent on several factors such as stage of the parasite, route and dose of administration and the genetic of the parasite (30). Dubey and Beattie revealed that the infection in intermediate hosts due to ingestion of the parasite oocysts were usually associated by more lesions in comparison to tachyzoites or tissue cysts (31).

Rat as an animal of choice for induction of chronic infections was shown to be infected even with only one live *Toxoplasma* tachyzoite (22). In several surveys on rats, the parasite was rarely noticed in histological sections of the infected animal with the RH strain (32, 33). In the study of Benedetto et al. (34), no tissue cysts were detected in brain of rats inoculated with the RH strain of the parasite. However, they showed that when the rats were treated with corticosteroids or were irradiated, clinical manifestations of toxoplasmosis were visible in the animals and small numbers of tissue cysts were found (34).

Moreover, it was clear that the passage of RH strain in rats resulted into attenuation of tissue cysts/bradyzoites (35). Tachyzoites of RH strain of the parasite could not lead to formation of tissue cysts in skeletal muscle (21). RH strain of the parasite lost its potential for formation of tissue cysts due to the prolonged passages of tachyzoites (26). For the first time, Sraei et al. (36) proposed that the Iranian strain has lost its ability of cystogenesis in rats.

Djurkovic-Djakovic et al. showed an association between the parasite and atovaquone and pyrrolidinedithiocarbamate for changing RH strain tachyzoites of *T. gondii* into tissue cysts in mice (23). The cysts of the RH strain detected in the brain of mice were shown not to be able to infect mice via the oral transmission because of previous treatment with sulfadiazine. Using immunofluorescence assays revealed no change for tachyzoite into bradyzoites (19).

The difference in pathogenicity of the RH strain in rats may be correlated with the genetic background of the parasite that happened during the prolonged passages in mice (37). Weiss and Kim reported a decrease in cyst formation in avirulent isolates of *T. gondii* due to prolonged *in vitro* passages of the parasite. They reported that it may be correlated with the rapid growths in the tissue culture (20). Also, the prolonged *in vitro* passages of other Apicomplexa such as *Besnoitia jellisoni* may result into the loss of the ability of tissue cyst formation in mice (18). In another study, it was demonstrated that S-48 as a live attenuated parasite strain was not able to produce any oocyst and tissue cyst which was reported to be a candidate for vaccines with commercialized veterinary purposes (38).

In our study, complementary tests were undertaken to evaluate the changes of tachyzoites into bradyzoites in cell cultures. To stimulate changing of tachyzoites into bradyzoites *in vitro*, various methods were employed such as altering the pH, addition of heat shock proteins, mitochondrial inhibitors or nitric oxide to the medium (39–41).

In our survey, cyst-like structures containing the organism stained with PAS were seen that may be due to a change in pH of cell culture media. PAS stains amylopectin granules and the presence of amylopectin granules in bradyzoites, rarely seen in tachyzoites, further supports the difference in consumption and metabolism of the carbohydrates (42). In our study, these organisms that were cultured in a particular condition were not resistant to acid-pepsin and the resistance to acid-pepsin explains this difference. However, some researchers presented opposite reports that oral infectivity in mice or acid-pepsin digestion cannot be considered as a crucial criterion (43, 44).

The results of numerous studies on tissue culture showed that bradyzoites spontaneously were converted into tachyzoites. The rate of conversion of bradyzoites into tachyzoites appears to be strain dependent (36, 45, 46). The high spontaneous rate of cyst formation in cell culture was due to the low virulence of the strains such as type II strain (39).

Isolation of *T. gondii* tissue cysts and bradyzoites from the cell cultures were undertaken employing few methods such as homogenization of the tissue, bradyzoite-specific antibody titers (39, 47), and isoenzyme patterns of glycolytic and tricarboxylic acid (TCA) cycle enzymes (48–51). Using molecular biology techniques, two forms of the parasite were discriminated due to expression of particular proteins such as surface antigens (52). For instance, mRNA expression of tachyzoite-specific SRS (SAG1- related sequences) was recognized in cyst forming strains due to immunesuppression in the infected mice (53).

Although Selseleh et al. could detect bradyzoite-specific surface antigens of BAG1 mRNA in the brain of mice infected by RH strain of the parasite and treated with sulfadiazine; they were not able to find any cyst in the mice brain by microscopic observation (54).

## Conclusion

This study confirmed that the RH strain of *T. gondii* has lost the potential for production of bradyzoite and tissue cyst forming in rats and bradyzoites cultivated in cell culture lost the most important criterion; the resistance to acidic condition, so this strain can be a candidate for future vaccine researches.
